# The prediction of influenza-like illness using national influenza surveillance data and Baidu query data

**DOI:** 10.1186/s12889-024-17978-0

**Published:** 2024-02-19

**Authors:** Su wei, Sun Lin, Zhao wenjing, Song Shaoxia, Yang Yuejie, He Yujie, Zhang Shu, Li Zhong, Liu Ti

**Affiliations:** 1 School of Management Science and Engineering, Shandong University of Finance and Economics, Jinan, Shandong 250014 People’s Republic of China; 2 Shandong Center for Disease Control and Prevention, Shandong Provincial Key Laboratory of Infectious Disease Control and Prevention, Shandong University Institution for Prevention Medicine, Jinan, Shandong 250014 People’s Republic of China; 3 Dezhou Center for Disease Control and Prevention, Dezhou, Shandong 253000 People’s Republic of China; 4 China Institute of Water Resources and Hydropower Research, Beijing, 100038 People’s Republic of China

**Keywords:** Influenza, Forecast, LSTM, Baidu search index

## Abstract

**Background:**

Seasonal influenza and other respiratory tract infections are serious public health problems that need to be further addressed and investigated. Internet search data are recognized as a valuable source for forecasting influenza or other respiratory tract infection epidemics. However, the selection of internet search data and the application of forecasting methods are important for improving forecasting accuracy. The aim of the present study was to forecast influenza epidemics based on the long short-term memory neural network (LSTM) method, Baidu search index data, and the influenza-like-illness (ILI) rate.

**Methods:**

The official weekly ILI% data for northern and southern mainland China were obtained from the Chinese Influenza Center from 2018 to 2021. Based on the Baidu Index, search indices related to influenza infection over the corresponding time period were obtained. Pearson correlation analysis was performed to explore the association between influenza-related search queries and the ILI% of southern and northern mainland China. The LSTM model was used to forecast the influenza epidemic within the same week and at lags of 1–4 weeks. The model performance was assessed by evaluation metrics, including the mean square error (MSE), root mean square error (RMSE) and mean absolute error (MAE).

**Results:**

In total, 24 search queries in northern mainland China and 7 search queries in southern mainland China were found to be correlated and were used to construct the LSTM model, which included the same week and a lag of 1–4 weeks. The LSTM model showed that ILI% + mask with one lag week and ILI% + influenza name were good prediction modules, with reduced RMSE predictions of 16.75% and 4.20%, respectively, compared with the estimated ILI% for northern and southern mainland China.

**Conclusions:**

The results illuminate the feasibility of using an internet search index as a complementary data source for influenza forecasting and the efficiency of using the LSTM model to forecast influenza epidemics.

## Background

Seasonal influenza and other respiratory tract infections remain serious public health problems. The WHO estimates that annual epidemics of influenza result in ~ 1 billion infections, 3 ~ 5 million severe cases of influenza and 300,000 ~ 650,000 deaths globally [1, 2]. A previous study estimated that 88,100 influenza-associated excess respiratory deaths occurred in China from 2010–2015 [3]. The National Health Commission of China reported that in 2020 and 2021, there were 1,145,278 and 668,246 influenza cases, with incidence rates of 81.5816 and 47.4008 per 100,000, respectively (http://www.nhc.gov.cn/jkj/s3578/202103/f1a448b7df7d4760976fea6d55834966.shtml, http://www.nhc.gov.cn/jkj/s3578/202204/4fd88a291d914abf8f7a91f6333567e1.shtml.). These conditions pose significant social and economic burdens in China. Due to the occurrence of coronavirus disease 2019 (COVID-19), the epidemic trend of influenza decreased rapidly in 2020. However, the intensity of the influenza epidemic has gradually increased since the spring of 2021. Thus, it is essential to establish an influenza surveillance system to monitor influenza epidemic trends. In China, the National Notifiable Infectious Disease Reporting System (NNIDRS) and hospital-based influenza surveillance system from the Chinese Influenza Center (CNIC) are used for surveillance communicable disease and for the surveillance of influenza or other respiratory viruses, respectively. The National Health Commission of China monthly reported the infectious disease data after one month, causing the influenza data to lag for one month, while the hospital-based surveillance system lagged for one or two weeks. Thus, it is necessary to establish a real-time influenza forecasting system to rapidly forecast influenza or other respiratory disease trends.

Currently, with the widespread use of the internet, people often seek help from the internet when they face health problems. When individuals search for information about health problems, including disease names, symptoms, therapies and prevention strategies, this information can be harnessed to monitor disease trends. In 2009, Ginsberg et al. first used Google query data to establish an influenza trend model to predict ILI rates in the U.S. in real time by monitoring millions of queries on their search engine; this approach overcomes the limitations of lag-time that are inherent to many traditional influenza surveillance systems [4]. Initially, the forecast system could provide accurate predictions and was expanded to other countries or regions. However, the forecast model was not stable because the influenza forecast trend exceeded the peak of the epidemic by more than 140% in 2013 in the U.S. and sparked a hot discussion about the limitation of search data in infectious disease research [5]. Since then, to predict disease epidemic trends, some research teams have attempted to assess the value of online search engines, including Google, Yahoo, Weibo, Baidu and Twitter, with different models and have obtained useful results. The field of digital epidemiology is still in an early stage, but it has begun to be used to forecast infectious disease epidemic trends, especially during the COVID-19 pandemic [6]. Thus, in the current article, we establish a forecasting model with Baidu search index data and ILI data to forecast trends in the incidence of seasonal influenza or other respiratory viruses in China.

The long short-term memory (LSTM) neural network is a model architecture for recurrent neural networks (RNNs) that has been widely applied in text classification, time series classification and time series forecasting [7, 8]. Using multilayer and complex neural networks close to real values, a backwards propagation algorithm is used to continually shrink the fitting error [9]. For infectious diseases, long short-term memory (LSTM) models, such as those for influenza and dengue, which have better accuracy, have been widely used in the prediction of different diseases and obtain good prediction results [10–14]. Previous research has confirmed that in the field of time series data analysis and prediction with complex relationships, LSTM in deep learning models yields better results than traditional machine learning methods [10, 15, 16]. In this paper, we incorporated the ILI and online search indices of different keywords into the LSTM model to forecast trends in influenza or other respiratory viruses and validate whether online search index data can improve forecasting accuracy.

## Materials and methods

### Data collection and processing

The weekly ILIs for both northern and southern mainland China were obtained separately from the Influenza Weekly Report published by the Chinese Influenza Center (CNIC) (http://ivdc.chinacdc.cn/cnic/) from January 2018 to December 2021. ILI patients were defined as outpatients of any age with acute respiratory infection syndrome with fever ≥ 38 °C and cough or sore throat. Influenza surveillance Sentinel hospitals distributed throughout all provinces of mainland China uploaded ILI case counts and total physician visit data to the Chinese Influenza surveillance system on Monday of the following week. The CNIC reports the aggregated data at the end of the next week, including the ILI%, which is the proportion of patients with ILI divided by the total number of physician visits. Due to the vast difference in the influenza epidemic situation among different regions, the CNIC releases the ILI% for northern and southern mainland China separately. The provinces in northern mainland China include Beijing, Tianjin, Hebei, Shanxi, Shaanxi, Inner Mongolia, Liaoning, Jilin, Heilongjiang, Shandong, Henan, Tibet, Gansu, Qinghai, Ningxia, and Xinjiang, and the provinces in southern mainland China include Shanghai, Jiangsu, Zhejiang, Anhui, Fujian, Jiangxi, Hubei, Hunan, Guangdong, Guangxi, Hainan, Chongqing, Sichuan, Guizhou, and Yunnan.

### Online Baidu index

Data on the Baidu search platform of 31 provinces were obtained from the Baidu Index (http://index.baidu.com) separately, an open online data service platform, on which we can obtain the daily search index for every keyword of every province. After filtering, we retrieved 30 keywords containing influenza-related symptom keywords or the keywords ‘flu’ or ‘influenza’ or the keywords of influenza prevention strategy for every province based on previous studies [4, 16, 17]. First, we collected the search indices of 30 selected influenza-related keywords from every province every day from 2018 to 2021 from the Baidu Index. Then, we obtained the weekly Baidu search index by adding the daily index. Finally, we computed the weekly index of the keywords northern and southern mainland China to obtain the total Baidu index of different keywords for northern and southern mainland China.

### Statistical analysis

#### Descriptive analysis

A descriptive analysis was used to reveal the characteristics of the current ILI% and the current search indices of different keywords on the Baidu search platform. The Pearson correlation coefficient was calculated to explore the association between the influenza-related search indices and the ILI% in northern and southern mainland China. We also analysed the correlation between the previous week's search index (from week 1 to 4) and the current ILI. A correlation coefficient closer to 1 or –1 indicates a stronger correlation, and a correlation coefficient closer to 0 indicates a weaker correlation. We calculated the Pearson correlation coefficient between each variable to observe the correlations between the variables. After performing the correlation analysis, we used the variables with correlation coefficients above 0.4 to develop forecasting models to improve the prediction accuracy based on previous studies [18, 19]. This study statistically analysed the usefulness of these potential predictors in forecasting ILI% and quantified their relationships during the influenza or respiratory illness seasons.

#### Module formulation

LTSM was used to predict the ILI% with the correlated search queries in China. LSTM is a special recurrent neural network (RNN) that is used to process sequence data. Compared with normal neural networks, RNNs perform well in processing sequential changes in data, but gradient disappearance and gradient explosion are inevitable. To solve this problem, an LSTM network is proposed for long sequence data, which has better performance than an RNN.

To implement information protection and control, there are an input gate, forget gate and output gate and a memory cell in each memory block.

The forget gate is controlled by a sigmoid to determine which information obtained from the previous moment can be retained at the current moment. The formula in the forget gate is F1. where ${W}_{f}$ is the weight matrix of the forget gate, ${x}_{t}$ is the current input, ${h}_{t-1}$ is the previous output of the memory block, ${b}_{f}$ is the bias term of the forget gate, and $\sigma$ is the sigmoid function.

1 $${f}_{t}=\sigma ({W}_{f}\cdot \left[{h}_{t-1},{x}_{t}\right]+{b}_{f})$$

The input gate decides how much information from the input ${x}_{t}$ can be reserved. The formula for the input gate is F2. Here, ${W}_{i}$ is the weight matrix of the input gate, ${b}_{i}$ is the bias term of the input gate, and the other parameters are the same as those of F1.

2 $${i}_{t}=\sigma ({W}_{i}\cdot \left[{h}_{t-1},{x}_{t}\right]+{b}_{i})$$

The output gate determines the degree of dependence of the input ${x}_{t}$ and the current memory cell. The formula for the input gate is F3. Here, ${W}_{o}$ is the weight matrix of the output gate, ${b}_{o}$ is the bias term of the output gate, and the other parameters are the same as those in F1.

3 $${o}_{t}=\sigma ({W}_{o}\cdot \left[{h}_{t-1},{x}_{t}\right]+{b}_{o})$$

In each *t,* there is a memory cell, and the cell state is important for LSTM, which allows the LSTM to select memory. The formulas for determining the cell state are F4, F5 and F6. where ${W}_{c}$ is the weight matrix of the current cell state, ${b}_{c}$ is the bias term of the current cell state, and $tanh$ is an active function.

4 $$\widetilde{{C}_{t}}=tanh({W}_{c}\cdot \left[{h}_{t-1},{x}_{t}\right]+{b}_{c})$$

5 $${C}_{t}={f}_{t}*{C}_{t-1}+{i}_{t}*\widetilde{{C}_{t}}$$

6 $${h}_{t}={o}_{t}*{{\text{tanh}}(C}_{t})$$

In this study, the absolute values of the Baidu index data and the ILI% were not on the same order of magnitude; we normalized all the data to be between 0 and 1 for further analysis and training. We defined the LSTM model with three layers, and there were 512 neurons in each layer. To reduce overfitting, we set a bias regularizer with regularization L2 (0.005). To train the model, we fit the model for 150 training epochs with a batch size of 64, and the learning rate was 0.0001. In the process, 126 sets of data (from week 201801 to week 202022) were used as the training set, 42 sets of data (from week 202023 to week 202111) were used as the validation set, and 41 sets of data (from week 202212 to week 202152) were used as the test set for model prediction. The obtained data were compared with the actual data to observe the model’s fitting effect. Moreover, to reduce overfitting, the dataset was augmented by averaging the data values of the two adjacent columns in turn and inserting the obtained average value between the two columns. Thus, the original dataset was expanded to eight times through three-time amplification. For northern and southern mainland China, both the ILI% and Baidu search indices were input into the LSTM module to train, validate and forecast, respectively. Four metrics were used to measure the performance of the LSTM model, namely, the R^2^, mean square error (MSE), root mean square error (RMSE) and mean absolute error (MAE), which measure the accuracy of a forecasting method in statistics. An R^2^ close to 1 and MAE and MSE close to 0 indicate the good prediction effect of the model. The RMSE is sensitive to extreme errors or very small errors in a set of measurements and can reflect the accuracy of the prediction.

## Results

### ILI% trend in China

**Fig. 1:**
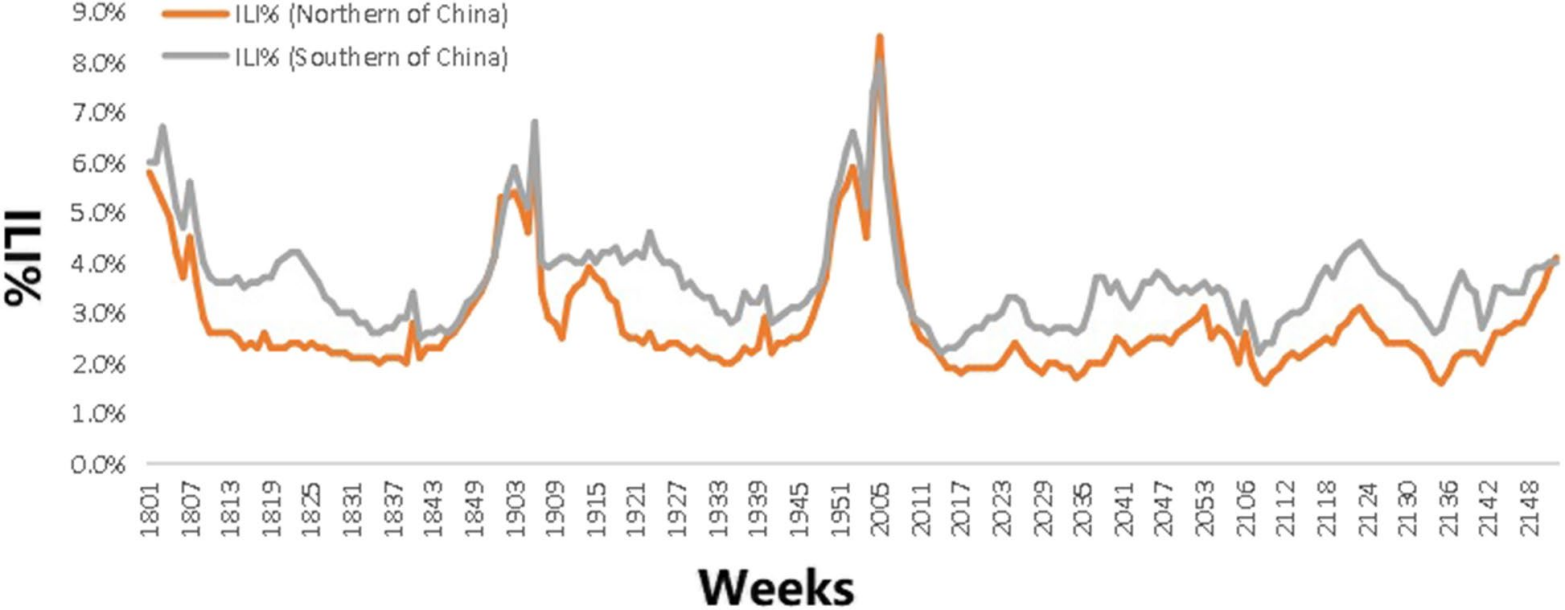
Different ILI% of Northern and Southern Mainland China. Note:1801 represents the first week in 2018

The ILI was reported every week of each year, and the ILI% presented a regular seasonal high incidence in northern and southern mainland China from 2018 to 2021. The average weekly ILI% was 2.79% and 3.62% in northern and southern mainland China, respectively. For northern mainland China, the highest ILI% was in the 1st week in 2018 (5.8%), the 6th week in 2019 (6.2%), the 5th week in 2020 (8.5%) and the 52nd week in 2021 (4.1%). For southern mainland China, the highest ILI% was observed in the 3rd week in 2018 (6.7%), the 6th week in 2019 (6.8%), the 5th week in 2020 (8.0%) and the 23rd week in 2021 (4.4%). During the period from January 2018 to March 2020, the highest ILI% was observed in the winter season in mainland China, and the duration of high ILI% was longer in the southern region than in the northern region; however, during the period from April 2020 to March 2021, the ILI% was lower than the average ILI%. Beginning in April 2021, the ILI% returned to its original level gradually, and two small peaks occurred in June and December 2021. (Fig. 1).

### Baidu search queries

We retrieved information on 30 search terms, including different influenza names, influenza symptoms, influenza drugs and mask sales. For all 30 terms, the weekly Baidu search index of northern and southern mainland China was calculated based on the diary search index of every province. The weekly average numbers of different keywords in the Baidu search indices for northern and southern mainland China are compared in Table 1. Pearson correlation analysis was also conducted between the Baidu search index and the ILI% across different lag periods, including the current week and a lag of one week, at lags of two, three and four weeks. The correlation coefficients of ILI% and different Baidu search queries varied widely in northern and southern mainland China. For northern mainland China, 24 terms of the Baidu search query statistics were correlated with the ILI% and with the lag weeks, with a correlation coefficient above 0.4, while only 7 terms were correlated in southern mainland China. The detailed information is provided in Table 1.

**Table 1:** Pearson association between Baidu search terms and ILI% in Northern and Southern mainland China from 2018–2021

Baidu search term	Northern mainland China						Southern mainland China					
	X ± S	The same week	lag one week	lag two weeks	lag three weeks	lag four weeks	X ± S	The same week	lag one week	lag two weeks	lag three weeks	lag four weeks
**Influenza Name**												
H1N1	10197 ± 7220	.545^**^	.547^**^	.547^**^	.548^**^	.551^**^	14509 ± 9684	.256^**^	.261^**^	.264^**^	.270^**^	.276^**^
H1N1 pdm2009 (甲流)	12346 ± 13810	.783^**^	.787^**^	.790^**^	.791^**^	.793^**^	15764 ± 13720	.701^**^	.711^**^	.721^**^	.729^**^	.734^**^
Liu xing xing gan mao (流行性感冒)	16987 ± 6280	.471^**^	.465^**^	.456^**^	.448^**^	.453^**^	14091 ± 6738	.003	.006	.006	.007	.009
Influenza (流感))	25118 ± 18581	.851^**^	.849^**^	.844^**^	.839^**^	.840^**^	36780 ± 24700	.704**	.700**	.693**	.680**	.678**
**Influenza Therapy**												
Compound amantadine tablet (感康)	21970 ± 5135	.528^**^	.523^**^	.517^**^	.515^**^	.518^**^	30114 ± 18819	-.028	-.023	-.018	-.012	-.005
Anti-cold drug (感冒药)	17535 ± 3082	.528^**^	.513^**^	.497^**^	.482^**^	.473^**^	24768 ± 11736	-.030	-.027	-.026	-.025	-.022
Ganmao Qingre Granules (感冒清热颗粒)	13625 ± 5285	.335^**^	.301^**^	.270^**^	.244^**^	.222^**^	18993 ± 12243	.005	.007	.008	.011	.014
What medicine to take for cold (感冒吃什么药)	16072 ± 10915	.371^**^	.380^**^	.388^**^	.397^**^	.405^**^	16347 ± 10314	.296^**^	.301^**^	.305^**^	.315^**^	.322^**^
Tamiflu (达菲)	14259 ± 8581	.803^**^	.794^**^	.812^**^	.820^**^	.816^**^	14047 ± 9661	.746^**^	.741^**^	.731^**^	.714^**^	.703^**^
Oseltamivir (奥司他韦)	27988 ± 30559	.833^**^	.827^**^	.832^**^	.839^**^	.836^**^	30232 ± 22107	.821^**^	.822^**^	.816^**^	.805^**^	.798^**^
Ostavir granules (奥司他韦颗粒)	10989 ± 10661	.644^**^	.635^**^	.625^**^	.616^**^	.605^**^	9341 ± 7248	.623^**^	.623^**^	.609^**^	.582^**^	.564^**^
White and black granules (白加黑)	13188 ± 2010	.602^**^	.586^**^	.570^**^	.560^**^	.554^**^	14552 ± 3897	.263^**^	.258^**^	.252^**^	.243^**^	.240^**^
Tylenol (泰诺)	13092 ± 2185	.726^**^	.714^**^	.703^**^	.693^**^	.687^**^	24620 ± 9757	.143^*^	.142^*^	.141^*^	.136	.136
**Influenza Symptom**												
Fever (发热)	11614 ± 2776	.510^**^	.517^**^	.524^**^	.530^**^	.538^**^	17681 ± 7523	.054	.061	.068	.078	.087
Heat (发烧)	24191 ± 7089	.862^**^	.859^**^	.857^**^	.855^**^	.852^**^	36299 ± 15463	.305^**^	.300^**^	.295^**^	.287^**^	.284^**^
Pneumonia (肺炎)	75549 ± 187776	.515^**^	.527^**^	.538^**^	.548^**^	.556^**^	108203 ± 203220	.342^**^	.351^**^	.360^**^	.375^**^	.385^**^
Wind chill Cold symptoms (风寒感冒症状)	11218 ± 5420	.166^*^	.175^*^	.182^**^	.191^**^	.197^**^	11665 ± 3675	.076	.086	.096	.111	.122
Cold and cough (感冒咳嗽)	11426 ± 4791	.487^**^	.470^**^	.444^**^	.419^**^	.398^**^	17292 ± 7640	.256^**^	.252^**^	.235^**^	.194^**^	.165^*^
Cold symptoms (感冒症状)	9713 ± 1947	.504^**^	.512^**^	.517^**^	.516^**^	.519^**^	14226 ± 6151	-.005	.003	.009	.016	.021
High fever (高烧)	8245 ± 1639	.819^**^	.811^**^	.804^**^	.799^**^	.794^**^	1642 ± 4097	.117	.120	.121	.118	.120
Catch cold (感冒)	29513 ± 7345	.623^**^	.609^**^	.597^**^	.587^**^	.584^**^	48640 ± 27627	.051	.051	.052	.051	.054
Cough (咳嗽)	34807 ± 8972	.703^**^	.692^**^	.682^**^	.673^**^	.668^**^	56864 ± 32017	.104	.104	.105	.104	.105
Sore throat (喉咙痛)	13635 ± 3920	.394^**^	.368^**^	.345^**^	.324^**^	.305^**^	36971 ± 12336	.333^**^	.325^**^	.316^**^	.305^**^	.297^**^
Symptoms of influenza A (甲型流感症状)	5027 ± 5543	.786^**^	.786^**^	.784^**^	.782^**^	.781^**^	6132 ± 5520	.713^**^	.718^**^	.721^**^	.722^**^	.721^**^
Influenza symptoms (流感症状)	12970 ± 13613	.847^**^	.844^**^	.838^**^	.837^**^	.833^**^	20076 ± 20104	.807^**^	.804^**^	.797^**^	.785^**^	.777^**^
Runny nose (流鼻涕)	7941 ± 4409	.108	.133	.156^*^	.179^**^	.200^**^	12986 ± 9310	-.052	-.039	-.024	-.004	.013
Headache (头痛)	15236 ± 1607	-.115	-.110	-.103	-.102	-.095	28202 ± 11522	-.184^**^	-.178^*^	-.171^*^	-.165^*^	-.159^*^
Pharyngalgia (咽痛)	5501 ± 1380	.420^**^	.428^**^	.433^**^	.435^**^	.442^**^	9190 ± 4061	-.005	.001	.006	.012	.019
**Influenza Prevention**												
Mask (口罩)	23254 ± 33162	.422^**^	.433^**^	.443^**^	.451^**^	.458^**^	35128 ± 48636	.228^**^	.236^**^	.244^**^	.257^**^	.267^**^
Surgical mask (医用外科口罩)	8882 ± 14,069	.423^**^	.434^**^	.443^**^	.452^**^	.459^**^	13252 ± 18928	.278^**^	.286^**^	.294^**^	.306^**^	.315^**^

x ± S Mean and standard deviation

^*^p < 0.05

^**^p < 0.001

### Evaluation scheme

**Fig. 2:**
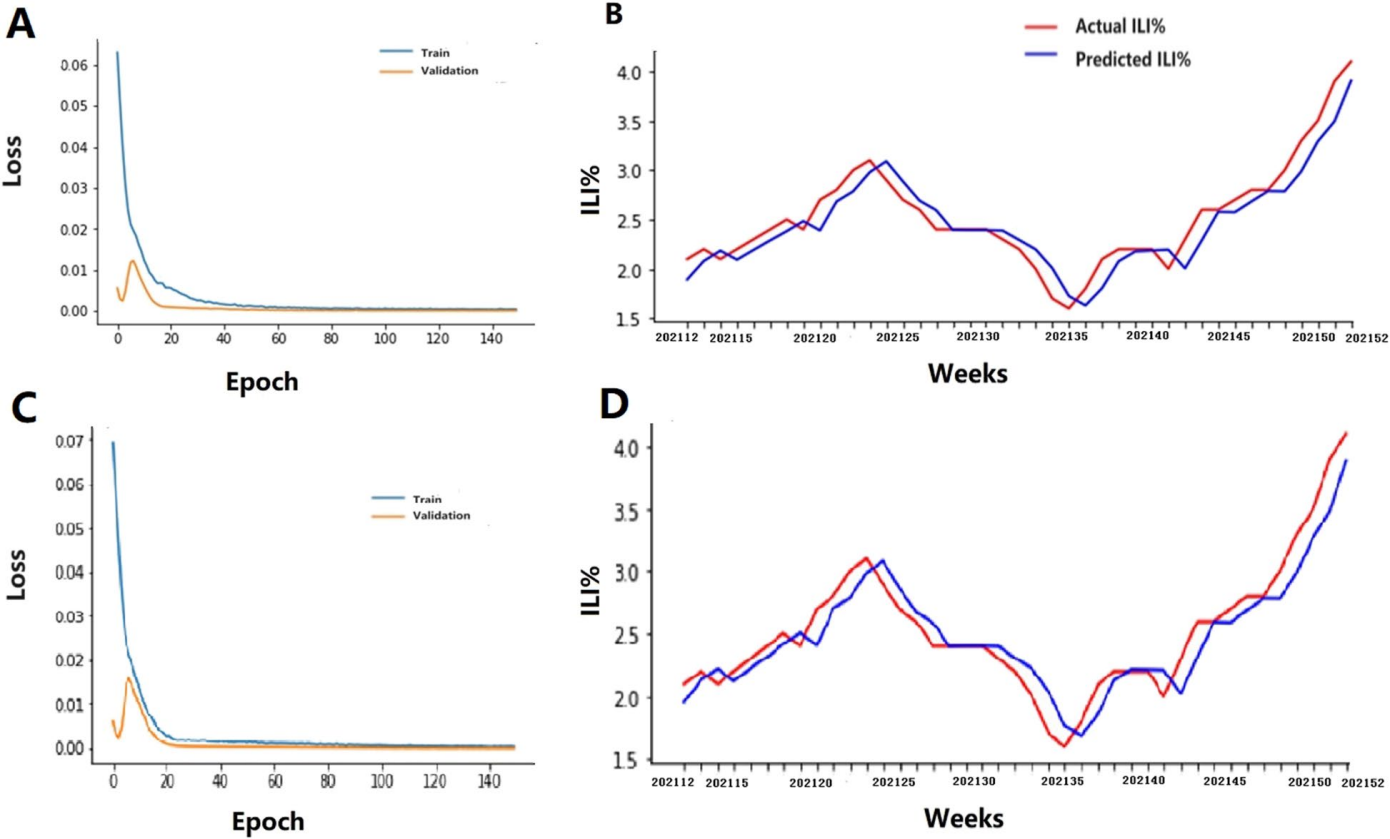
Actual and predicted ILI% of Northern mainland China in 2021. **A** Training and Validation of LSTM model with ILI%. **B** Actual and predicted ILI% with ILI%. **C** Training and Validation of LSTM model with ILI% + mask with one lag week. **D** Actual and predicted ILI% with ILI% + influenza + mask with one lag week. Note: 202112 represents the twelfth week in 2021

**Fig. 3:**
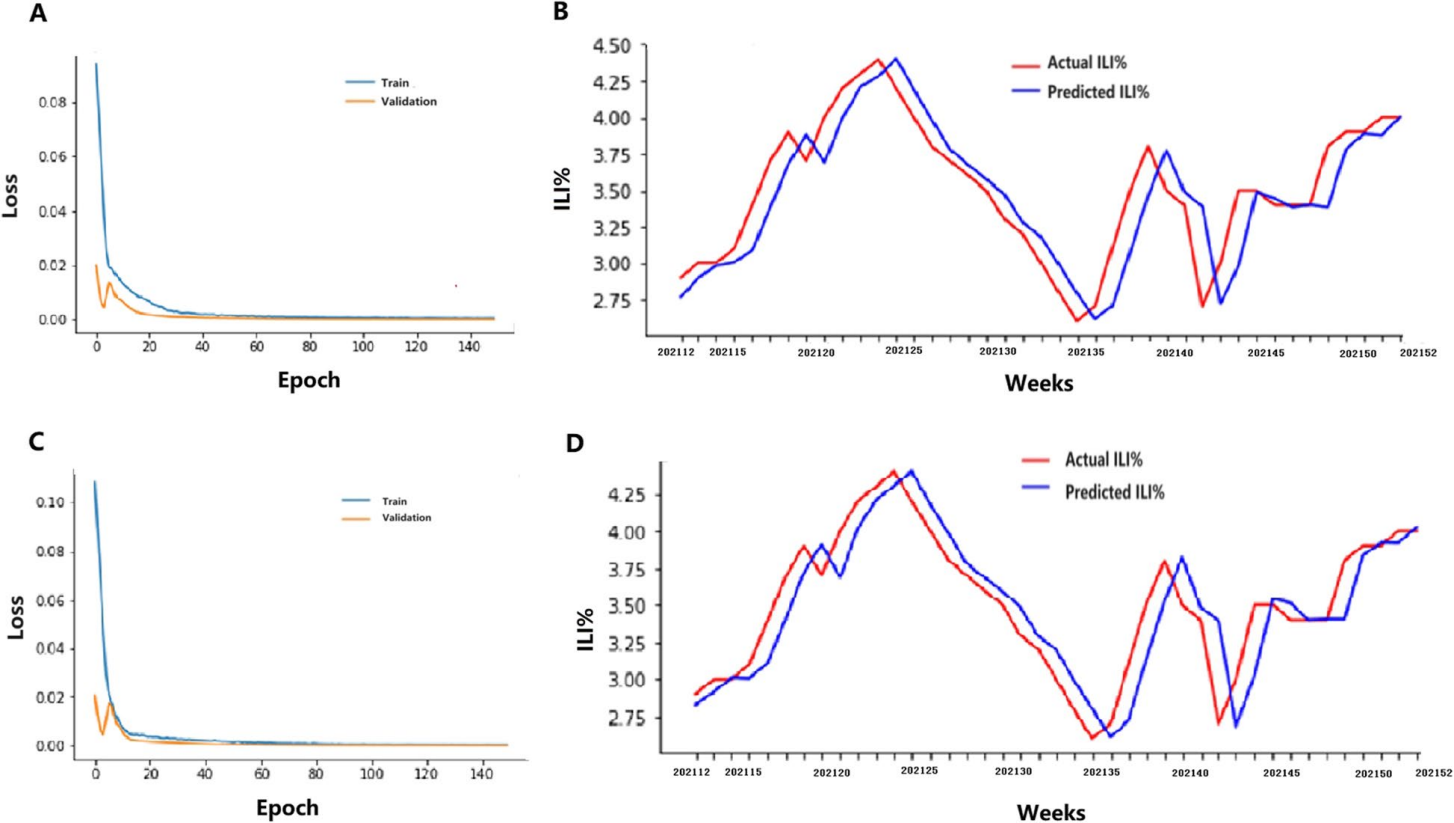
Actual and predicted ILI% of Southern mainland China in 2021. **A** Training and Validation of LSTM model with ILI%. **B** Actual and predicted ILI% with ILI%. **C** Training and Validation of LSTM model with ILI% + influenza name. **D** Actual and predicted ILI% with ILI% + influenza name. Note: 202112 represents the twelfth week in 2021

First, the original ILI% was solely input into the LSTM module as the gold standard for predicting the ILI% activity trend. Second,, the ILI% was simultaneously input into the LSTM module with a Baidu search index with a correlation coefficient above 0.4; these factors were divided into five categories with different lag times – namely, ILI% + all of the Baidu index, ILI% + the index of influenza name, ILI% + the index of influenza therapy and drug, ILI% + the index of influenza symptoms and ILI% + the index of mask – to compare the effects of the different combinations with the calculated MSE, RMSE, MAE and R^2^. For northern mainland China, the R^2^ of ILI% + the index of masks with one lag week module reached 0.9055, which was greater than the corresponding values of the ILI% alone, and other combinations of ILI% + the Baidu search index. Similarly, the MAE was 0.14325, and the MSE was 0.02762, which were lower than the corresponding values of the ILI% alone, the other combinations of ILI% + the Baidu search index (Table 2). For southern mainland China, the R^2^ of ILI% + the index of the influenza name module reached 0.75579, which was higher than the corresponding values of the ILI% alone, other combinations of ILI% + the Baidu search index. Similarly, the MAE was 0.17832, and the MSE was 0.05211, which were lower than the corresponding values of the ILI% alone, and other combinations of ILI% + the Baidu search index(Table 2). These results showed that ILI% + the index of masks with one lag week and ILI + the index of influenza name had the best prediction effects for northern and southern mainland China, respectively. The LSTM module reduced the RMSE predictions by 16.75% and 4.20% compared with the estimated ILI% for northern and southern mainland China, respectively. We then constructed a prediction diagram, and the results showed that the actual values were consistent with each other and that the accuracy was high (Figs. 2 and 3).

**Table 2:** The different Metric of different Baidu search Index and ILI% with LSTM

Catagories	Northern Mainland China				Southern Mainland China			
	MSE	RMSE	MAE	R2	MSE	RMSE	MAE	R2
**ILI%**	**0.0399**	**0.19965**	**0.15586**	**0.86066**	**0.05678**	**0.23828**	**0.18511**	**0.73390**
ILI% + all baidu index	0.08767	0.29609	0.25347	0.67951	0.05864	0.24216	0.18526	0.72516
lag1	0.04725	0.21736	0.18197	0.82728	0.05607	0.23679	0.19064	0.73720
lag2	0.03801	0.19497	0.15378	0.86419	0.05767	0.24016	0.19341	0.72437
lag3	0.03804	0.19504	0.14516	0.86094	0.05250	0.22913	0.17980	0.75394
lag4	0.04199	0.20491	0.15761	0.85321	0.05681	0.23835	0.18624	0.72849
ILI% + Influenza name	0.03056	0.17482	0.14430	0.88827	**0.05211**	**0.22827**	**0.17832**	**0.75579**
lag1	0.03233	0.17982	0.14516	0.88180	0.05373	0.23180	0.18075	0.74817
lag2	0.06729	0.25941	0.21462	0.75400	0.05686	0.23846	0.18900	0.73349
lag3	0.11286	0.33595	0.28441	0.59075	0.05580	0.23621	0.18446	0.73849
lag4	0.13701	0.37016	0.32162	0.49913	0.05512	0.23478	0.18232	0.74165
ILI% + influenza symptom	0.04411	0.21003	0.17060	0.83874	0.05638	0.23744	0.18353	0.73577
lag1	0.03908	0.19769	0.15593	0.85828	0.05380	0.23195	0.17896	0.74785
lag2	0.03464	0.18612	0.14976	0.87624	0.05381	0.23197	0.18338	0.74779
lag3	0.02983	0.17271	0.13802	0.89096	0.05659	0.23789	0.18571	0.73476
lag4	0.03173	0.17813	0.14395	0.88400	0.05453	0.23351	0.18047	0.74443
ILI% + influenza therapy	0.03407	0.18458	0.15026	0.87545	0.05267	0.22950	0.18412	0.75399
lag1	0.04266	0.20655	0.16336	0.84530	0.05797	0.24078	0.19197	0.72828
lag2	0.04286	0.20703	0.16656	0.84332	0.05583	0.23629	0.18675	0.73831
lag3	0.04395	0.20964	0.17098	0.83934	0.05400	0.23239	0.18195	0.74689
lag4	0.03025	0.17391	0.13922	0.88943	0.05487	0.23425	0.18095	0.74281
ILI% + Mask	0.03116	0.17651	0.14589	0.88611				
**lag1**	**0.02762**	**0.16620**	**0.14325**	**0.90554**				
lag2	0.03136	0.17710	0.14650	0.88535				
lag3	0.03076	0.17539	0.14474	0.88754				
lag4	0.03164	0.17788	0.14707	0.88433

## Discussion and conclusion

The dynamic assessment and forecasting of epidemic trends are important parts of the prevention and control of infectious diseases. The ILI% is a good indicator for detecting respiratory illness and influenza viruses. To predict respiratory illness trends, the ILI% was used to predict trends in influenza virus or respiratory illness incidence. To predict the trend precisely, several researchers have used the ILI% and search indices to predict respiratory illness incidence via different methods, such as the seasonal autoregressive integrated moving average (SARIMA) model and linear regression models [20–23]. However, the results have shown that the prediction accuracy is not high [16, 24]. With the development of artificial intelligence, machine learning algorithms have shown advantages in prediction and recognition. LSTM is an advanced RNN with the ability to learn time patterns and store useful memories longer. This type of LSTM has been widely used to analyse and predict time series data in various sectors and was confirmed to outperform some statistical-based algorithms. [10, 25]. At present, there are few reports on the prediction of influenza infection with an RNN combined with the Baidu Index [16]. In this study, we reviewed the Baidu search index related to ILI% and proposed an LSTM model to predict the occurrence of respiratory disease or influenza virus in northern and southern mainland China; the results confirmed that the Baidu search intensity of keywords is a useful disease surveillance tool and further showed that the ILI% + Baidu search index performed significantly well as a predictor compared with the ILI% alone.

Previous research on disease predictions has shown that data from social media, including Google, Twitter and other media containing important information, can be used to effectively predict disease incidence, and there is a strong correlation between disease searchers and disease cases [26–30]. Moreover, the search behaviour of the user could show the degree of concern of the user to a certain event or something. Information search behaviour is a targeted information acquisition behaviour carried out by users to meet their specific needs [31]. When people around them suffer from influenza or have influenza-like symptoms, many people tend to search for influenza prevention measures, flu symptoms and other related information from the internet. In this study, our results revealed that 7 Baidu search queries strongly correlated with ILI%, not only in northern mainland China but also in southern mainland China; these queries included H1N1 pdm2009, influenza, Tamiflu, oseltamivir, oseltamivir granules, symptoms of influenza A, and symptoms of influenza. However, in northern mainland China, the other 17 items still had a strong correlation with the ILI%. To pursue this reason, some possible intrinsic limitations in the application of search data for epidemic disease surveillance should be considered. For instance, web users’ educational level, regional background, cognition level and disease epidemic trends can influence users’ search habits and keywords. For influenza, there is only one epidemic peak annually in northern mainland China, while there are two epidemic peaks in southern mainland China. Differences in influenza epidemic trends may influence cognition levels and thus influence behavioural habits. In northern mainland China, most people suffer from influenza or respiratory illness in winter; thus, the search index increases rapidly in winter, including some symptoms and use of masks. However, in southern mainland China, most people suffer from influenza or respiratory illness twice a year [32]; thus, they learn about epidemic trends; thus, they do not focus on symptoms or prevention measures. Therefore, we cannot integrate all the search indices into the model to forecast disease epidemic trends because not all Baidu indices are strongly correlated with the ILI%. According to the LSTM model, the ILI% + mask at lag 1 week was a good predictor of the ILI% trend in northern mainland China; however, for southern China, the ILI% + influenza name was the best predictor, which has not been discussed in other studies. Therefore, for disease prediction, high correlation data and classification data can improve the accuracy, and some classification data further strengthen the prediction accuracy. Our study revealed that geographical location may affect the prediction of disease epidemics.

The algorithms and computational techniques used for computation and analysis still need to be carefully refined, tuned and calibrated to avoid overfitting risk in big data. To avoid overfitting issues in the LSTM model, in our study, after three amplifications, the data (including one ILI sequence and twenty-four or seven Baidu Index sequences) were found to be sufficient to improve the robustness of our training effect at the data level. Second, the LSTM model is a lightweight and appropriate model for solving our target problem. This model provides several methods for reducing overfitting, including increasing the number of LSTM layers, increasing the number of LSTM units, eliminating dropouts, using regularization, and using additional training data. In our paper, to reduce overfitting, we modified the LSTM model by adding three layers and 512 units in each layer and used biasregularizer to train the model.

The prediction of epidemic trends due to influenza or respiratory illness is a topic of intense discussion worldwide. Adding the Baidu search index of influenza-related keywords to influenza forecasting can effectively improve the accuracy of influenza forecasting in China. Furthermore, the influence of the search indices of different keywords on the accuracy of the prediction results varies. The next step of this research will involve incorporating relevant meteorological factors into the model, hoping to construct more accurate prediction models of influenza and respiratory diseases through multidimensional factors.

## Data Availability

The ILI% weekly data for the study was search to obtain from Chinese National Influenza Center (http://ivdc.chinacdc.cn/cnic/) and the weekly Baidu query data was searched from Baidu search index (https://index.baidu.com/v2/index.html#/), the datasets are available from the corresponding author on reasonable request.
